# Association of RDW–Albumin Ratio, TG-Glucose Index, and PIV with Coronary Artery Disease

**DOI:** 10.3390/jcm13237003

**Published:** 2024-11-21

**Authors:** Emre Akkaya

**Affiliations:** Department of Cardiology, Bossan Hospital, Gaziantep 27580, Turkey; dremreakkaya@hotmail.com; Tel.: +90-505-656-35-87

**Keywords:** coronary artery disease, red blood cell distribution width–albumin ratio, triglyceride–glucose index, pan-immune-inflammation value, prediction

## Abstract

**Objectives:** This study aimed to investigate the impact of the RDW–albumin ratio (RAR), Triglyceride–glucose index (TGI), and pan-immune-inflammation value (PIV) on predicting prognosis in patients with coronary artery disease (CAD) and to assess the potential use of these biomarkers in clinical decision-making. **Materials and Methods:** This retrospective study involved patients diagnosed and treated from 2020 to 2024. The study population included individuals diagnosed with CAD (n = 450) as well as a control group without CAD (n = 150). **Results:** The RAR, TGI, and PIV were significantly higher in the CAD group (*p* < 0.01 for all). Furthermore, a high RAR was found to be a risk factor for CAD (OR = 1.4, 95% CI: 1.2–1.7, *p* < 0.01), while elevated TGI was also linked to an increased risk of CAD (OR = 1.5, 95% CI: 1.3–1.8, *p* < 0.01). Similarly, a high PIV was strongly associated with CAD risk (OR = 2.0, 95% CI: 1.7–2.4, *p* < 0.01). The combined analysis of RAR, TGI, and PIV yielded an AUC value of 0.78 (0.75–0.81), indicating that these biomarkers collectively provide high diagnostic accuracy for CAD, with a sensitivity of 74% and specificity of 77% (*p* < 0.01). **Conclusions:** In conclusion, our study not only emphasizes the significance of traditional risk factors in CAD, but also highlights new biomarkers that could improve predictive accuracy. The combined use of biomarkers such as the RAR, TGI, and PIV offers greater accuracy in diagnosing CAD. Thus, our research presents an innovative approach with the potential to enhance the prediction and management of CAD risk.

## 1. Introduction

Cardiovascular diseases (CVD) were responsible for approximately 17.9 million deaths in 2019, with 85% of these deaths attributed to heart attacks and strokes. Coronary artery disease (CAD) constitutes a significant portion of these fatalities [1]. CAD is a major cause of death globally and is characterized as an atherosclerotic disease, where inflammation plays a key role. Alongside inflammatory responses, metabolic and hematological parameters are also known to contribute to CAD pathogenesis [2]. In this regard, the use of biomarkers for predicting prognosis and treatment outcomes in CAD is becoming increasingly important.

The red blood cell distribution width (RDW) to albumin ratio (RAR) is considered an important indicator of inflammatory response and nutritional status [3]. Elevated RDW levels can generally indicate chronic inflammation and nutritional deficiencies, suggesting cellular stress and anemia in the body [4]. Conversely, low albumin levels have been linked to cardiovascular diseases and an increased risk of mortality. Albumin is a protein that plays vital roles in protein transport and maintaining osmotic balance. Its low levels can result from inflammation, malnutrition, or chronic diseases [5].

The Triglyceride–glucose index (TGI) is a crucial biomarker used to assess insulin resistance, combining plasma triglyceride and fasting blood glucose levels to reflect insulin sensitivity [6]. Elevated TGI levels have been related with conditions such as diabetes mellitus and metabolic syndrome, and are considered a risk factor for cardiovascular diseases [7]. This parameter indicates disruptions in lipid metabolism and glucose homeostasis and may play a significant role in the pathogenesis of CAD.

The pan-immune-inflammation value (PIV) is a composite marker that provides an overall assessment of inflammatory status, incorporating various immune and inflammatory components to reflect the total inflammatory burden and immune system activity [8]. PIV can help determine the extent of chronic inflammation, which plays a critical role in the development of systemic conditions such as atherosclerosis, a key contributing factor to cardiovascular diseases like CAD. High PIV levels indicate an increased inflammatory response and potential cardiovascular risks [9].

This study aims to investigate the impact of the RAR, TGI, and PIV on prediction in patients with CAD, and to evaluate how these biomarkers can be utilized in clinical decision-making.

## 2. Materials and Methods

### 2.1. Study Design and Study Population

Patients were enrolled through a retrospective analysis of medical records from the Department of Cardiology, Bossan Hospital, Gaziantep, between 2020 and 2024. All patients who underwent elective coronary angiography or Coronary Computed Tomography Angiography (CCTA) for stable coronary artery disease (CAD) during this period were screened for inclusion. Patients were included in the study if they were 18 years or older and had a confirmed diagnosis of CAD, defined as at least one coronary artery with a stenosis of 50% or more. None of the patients were in the acute phase of CAD, such as acute myocardial infarction or unstable angina, at the time of angiography. Enrollment for coronary angiography was based on the following criteria:

✓ Chronic stable angina—Patients with persistent chest pain or anginal symptoms occurring during exertion or emotional stress and relieved by rest or medication;
✓ Abnormal non-invasive test results—Patients with positive findings from stress tests (e.g., treadmill exercise, stress echocardiography, myocardial perfusion imaging) that indicated myocardial ischemia;
✓ Intermediate or high-risk profiles—Patients with risk factors such as diabetes mellitus, hypertension, hypercholesterolemia, or a family history of CAD who had symptoms suggestive of CAD.

Individuals presenting with acute coronary syndromes (ACS) or lacking angiographic confirmation were excluded from the study to avoid misclassification. The control group consisted of patients who underwent cardiac evaluation during the same period but were found not to have significant coronary artery disease, based on either normal angiography or non-significant stenosis (<50%).

Patients with more than 10% missing data in their medical records were excluded to maintain data integrity. A total of 450 CAD patients and 150 controls were included in the final analysis.

Data were collected from the hospital’s medical records, including sociodemographic information, clinical characteristics, and laboratory findings. The key variables analyzed included age, gender, body mass index (BMI), smoking status, hypertension, hypercholesterolemia, diabetes mellitus, and ejection fraction (EF). Blood samples were collected and analyzed to determine the levels of hemoglobin, red blood cell distribution width (RDW), leukocytes, neutrophils, lymphocytes, monocytes, platelets, glucose, blood urea nitrogen (BUN), total cholesterol, HDL, LDL, triglycerides, bilirubin, creatinine, and albumin.

RDW–albumin ratio (RAR) was calculated by dividing the RDW percentage by the albumin level. Triglyceride–glucose index (TGI) was evaluated utilizing the formula [Ln (triglyceride level (mg/dL) × glucose level (mg/dL)/2)]. Pan-immune-inflammation value (PIV) was determined by combining the counts of monocytes, neutrophils, platelets, and lymphocytes, with the formula that monocyte count × neutrophil count × platelet count/lymphocyte count.

### 2.2. Statistical Analysis

Statistical analysis was conducted using SPSS software (version 27.0, IBM, SPSS Inc., Chicago, IL, USA). Demographic and clinical characteristics were described as mean and standard deviation for continuous variables and as frequencies and percentages for categorical variables. Descriptive statistics, *t*-tests, and chi-square tests were used for group comparisons. Prior to conducting *t*-tests, the normality of continuous variables was assessed using the Shapiro–Wilk test, and non-parametric tests were applied for non-normally distributed data. Stepwise logistic regression analysis (forward method) was employed to evaluate the relationship between the parameters and CAD, with variables selected based on their univariate association (*p* < 0.1) to prevent overfitting and multicollinearity. Receiver operating characteristic (ROC) curve analysis was performed to determine sensitivity, specificity, and cut-off values for predicting CAD, and comparisons between ROC curves were conducted using the DeLong test to assess improvements in model performance.

## 3. Results

The comparison of sociodemographic and clinical characteristics is shown in Table 1. The mean age of patients in the CAD group (70.3 ± 9.8 years) was found to be higher than that of the normal group (65.0 ± 10.3 years) (*p* < 0.02). The proportion of male patients in the CAD group was 60%, while this proportion was 50% in the normal group (*p* < 0.01). In terms of BMI, DBP, SBP, heart rate, and EF, there was a statistically significant increase in patients with CAD compared to the control group (*p* < 0.04, *p* < 0.03, *p* < 0.03, *p* < 0.03, and *p* < 0.001, respectively). In terms of smoking rate, hypertension, hypercholesterolemia, and diabetes mellitus, there was a statistically significant increase in patients with CAD compared to the control group (*p* < 0.001) (Table 1).

A comparison of laboratory findings is shown in Table 2. In terms of leukocyte, neutrophils, monocytes, and RDW values, compared to the control group, there was a statistically significant increase in patients with CAD (*p* < 0.05). In terms of platelets, glucose, total cholesterol, LDL and triglyceride values, compared to the control group there was a statistically significant increase in patients with CAD (*p* < 0.01). In terms of bilirubin, creatinine, and BUN values, compared to the control group there was a statistically significant increase in patients with CAD (*p* < 0.05). In terms of hemoglobin, lymphocytes, HDL, and albumin levels, there was a statistically significant decrease in patients with CAD compared to the control group (*p* < 0.05, *p* < 0.05, *p* < 0.01, and *p* < 0.01, respectively). RAR (4.0 ± 0.7) in the CAD group was statistically higher than in the normal group (3.2 ± 0.5) (*p* < 0.01). TGI (5.0 ± 0.8) in the CAD group was statistically higher than in the normal group (4.2 ± 0.6) (*p* < 0.01). PIV (500 ± 150) in the CAD group was statistically higher than in the normal group (250 ± 100) (*p* < 0.01) (Table 2).

The stepwise logistic regression analysis (forward method) of factors used for patients with CAD is shown in Table 3. The results demonstrate that age was a significant predictor, with each additional year increasing the odds of CAD (OR = 1.04, 95% CI: 1.02–1.06, *p* < 0.01). Similarly, male gender was associated with a risk of CAD (OR = 1.1, 95% CI: 0.8–1.2, *p* < 0.01). Elevated body mass index (BMI) also emerged as a significant factor, with higher BMI values correlating with increased odds of CAD (OR = 1.2, 95% CI: 1.1–1.3, *p* < 0.01). Smoking further amplified CAD risk, with smokers having a significantly higher likelihood of developing the condition (OR = 2.0, 95% CI: 1.4–2.8, *p* < 0.01). Among clinical conditions, hypertension and hypercholesterolemia were strongly associated with CAD, with odds ratios of 2.5 (95% CI: 1.8–3.4, *p* < 0.01) and 1.8 (95% CI: 1.5–2.3, *p* < 0.01), respectively. Diabetes mellitus was another critical predictor, doubling the risk of CAD (OR = 2.0, 95% CI: 1.5–2.8, *p* < 0.01). Ejection fraction (EF), a measure of cardiac function, showed an inverse relationship with CAD risk (OR = 0.9, 95% CI: 0.8–0.9, *p* < 0.01), indicating that lower EF values are linked to higher CAD likelihood. Additionally, higher neutrophil counts and lower HDL levels were significantly associated with CAD, underscoring their roles in the inflammatory and lipid profiles of patients. Novel biomarkers also played a significant role in CAD risk prediction. Elevated red blood cell distribution width (RDW) and a higher RDW–albumin ratio (RAR) were both strong predictors (OR = 1.2, 95% CI: 1.1–1.4, *p* < 0.01; OR = 1.4, 95% CI: 1.2–1.6, *p* < 0.01, respectively). The triglyceride glucose index (TGI), a marker of insulin resistance, was linked to an increased CAD risk (OR = 1.6, 95% CI: 1.3–2.0, *p* < 0.01). Furthermore, the pan-immune-inflammation value (PIV), which reflects systemic inflammatory burden, emerged as a strong predictor of CAD (OR = 2.0, 95% CI: 1.7–2.4, *p* < 0.01). These findings highlight the complex interplay of traditional and novel biomarkers in the pathogenesis of CAD and their potential utility in improving risk stratification in clinical practice (Table 3).

**Figure 1:**
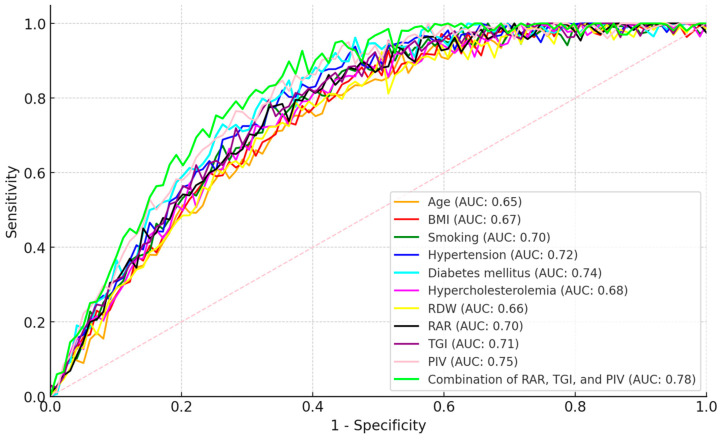
ROC analysis results in patients with CAD.

The results of ROC analysis in patients with CAD are shown in Table 4. The AUC value for age was 0.65 (0.62–0.68), indicating that age is a moderate determinant in the diagnosis of CAD. Sensitivity was 60%, specificity was 65%, and the cut-off value was 65 years and above (*p* < 0.01). The AUC value for BMI was 0.67 (0.64–0.70), indicating that BMI is effective in the diagnosis of CAD. Sensitivity was 63%, specificity was 68%, and the cut-off value was 27.5 kg/m^2^ and above (*p* < 0.01). The AUC values for smoking, hypertension, diabetes mellitus, and hypercholesterolemia are close to each other (AUC: 0.68–0.72), indicating that these parameters are strong risk factors in the diagnosis of CAD (*p* < 0.01). The AUC value for RDW was 0.66 (0.63–0.69), indicating that RDW plays a certain role in the diagnosis of CAD. Sensitivity was 64%, specificity was 66%, and the cut-off value was 14.5% and above (*p* < 0.01). The AUC value for RAR was 0.70 (0.67–0.73), indicating that RAR is significant in the diagnosis of CAD. Sensitivity was 68%, specificity was 70%, and the cut-off value was 3.8 and above (*p* < 0.01). The AUC value for TGI was 0.71 (0.68–0.74), indicating that TGI is an important determinant for CAD. The sensitivity was 69%, the specificity was 71%, and the cut-off value was 9.0 and above (*p* < 0.01). The AUC value for PIV was 0.75 (0.72–0.78), indicating that PIV can be used with high accuracy in the diagnosis of CAD. The sensitivity was 72%, the specificity was 75%, and the cut-off value was 368.8 and above (*p* < 0.01). The AUC value for the combination of RAR, TGI and PIV was 0.78 (0.75–0.81), indicating that the combination of these three parameters provides a very strong determination in the diagnosis of CAD. The sensitivity was 74%, and the specificity was 77% (*p* < 0.01) (Table 4, Figure 1).

## 4. Discussion

Our study provides significant contributions to the literature by evaluating the use of new biomarkers such as RAR, TGI and PIV in the diagnosis of CAD. This study addresses for the first time the combined use of RAR with TGI and PIV in the diagnosis of CAD. In particular, the ROC analysis results obtained in our study show that this combination offers higher specificity and sensitivity in the diagnosis of CAD. These findings are consistent with those from the studies in the literature examining the prognostic role of RAR in other diseases, but such a comprehensive evaluation in the context of CAD is rare. Other studies have generally examined RAR alone or with a limited number of biomarkers, whereas our study reveals how the combined use of these biomarkers can increase diagnostic accuracy.

High leukocyte counts have been related with an increased risk of atherosclerosis and cardiovascular diseases, including CAD. Elevated leukocyte counts contribute to inflammation, which plays an important role in the development and progression of atherosclerosis [10]. Studies have shown that individuals with higher leukocyte counts are at an elevated risk for various cardiovascular conditions, including ischemic heart disease and myocardial infarction [10,11]. Neutrophils, a type of white blood cell, are particularly implicated in the pathogenesis of atherosclerosis. High neutrophil counts have been linked to the promotion of plaque formation and instability, leading to increased cardiovascular risk. Neutrophils contribute to the oxidative modification of LDL cholesterol, which is a key step in atherogenesis [11]. Recent studies have demonstrated that higher neutrophil counts are associated with an increased risk of cardiovascular diseases, including CAD [10,11]. Overall, our findings corroborate the established literature, underscoring the significance of leukocyte and neutrophil counts as markers of inflammation in CAD.

Recent studies have increasingly focused on the RAR as a potential biomarker for predicting adverse outcomes in cardiovascular diseases, including CAD [12,13,14,15]. The RAR integrates measures of inflammation and nutritional status, providing a more comprehensive risk assessment. Research has shown that a higher RAR is associated with worse outcomes in various cardiovascular conditions, such as acute myocardial infarction (AMI) and diabetic ulcers [14]. In a study of Li et al., it was shown that patients with higher RAR levels had significantly higher 90-day mortality rates post-AMI, indicating that RAR is a strong predictor of short-term mortality in these patients [16]. Similarly, in a study of Hong et al., it was shown that there was an association between high RAR and increased all-cause mortality in patients with type 2 diabetes and Diabetic Foot Ulcers (DFUs), reinforcing the marker’s prognostic utility across different patient populations [15]. The association of RAR with poor cardiovascular outcomes is believed to be due to the dual impact of elevated RDW, which reflects variability in red blood cell size (often associated with inflammation and oxidative stress), and low albumin levels, indicative of poor nutritional and inflammatory status. This combination is thought to exacerbate endothelial dysfunction and promote atherosclerosis, leading to worse outcomes in CAD [12,17]. Our study builds on these findings by specifically examining the role of RAR in a cohort of CAD patients, providing additional evidence for its use as a predictive biomarker. Unlike previous studies that often focused on single conditions or broad cardiovascular outcomes [12,13,14,15,16], our research offers a detailed analysis of RAR’s role in CAD, including its correlation with other well-known risk factors and novel biomarkers such as the TGI and PIV.

Recent studies have reported that an elevated TGI is significantly related with increased risks of adverse cardiovascular events, such as coronary artery stenosis, major adverse cardiovascular events (MACEs), and the severity of CAD [18,19,20]. The TGI has been shown to correlate with the complexity and severity of CAD. In a study of Xiong et al., it was reported that patients with higher TGI levels had a significantly increased risk of severe CAD, as indicated by a higher SYNTAX score, which is used to assess the complexity of coronary artery lesions [18]. In a study of Gao et al., they found higher TGI levels with a greater incidence of coronary artery calcification and poor coronary collateralization in patients with chronic total occlusion (CTO) lesions, indicating more advanced disease and worse prognosis [21]. The relationship between TGI and MACEs has been explored, particularly in patients with conditions like hypertension and diabetes, which exacerbate cardiovascular risk. In a study by Tao et al., it was stated that higher TGI levels predict a higher likelihood of MACEs, underscoring the index’s utility in identifying patients at high risk of recurrent cardiovascular events [22]. Our study adds to this body of literature by specifically examining the role of TGI in a cohort of CAD patients and integrating it with other novel biomarkers such as RAR and PIV. This approach offers a more nuanced understanding of CAD risk stratification and highlights the potential of TGI as a significant predictor of CAD outcomes.

PIV combines counts of neutrophils, monocytes, lymphocytes, and platelets, reflecting the systemic inflammatory and immune response status of the body. Recent studies have demonstrated its predictive value in CAD, particularly in assessing the severity of the disease and predicting adverse cardiovascular events [23,24,25]. In a study of Dolapoglu et al., it was reported that higher PIV levels were independently related with an increased risk of MACCE. The study demonstrated that PIV was a better predictor of MACCE compared to traditional markers like the Systemic Immune-Inflammation Index (SII) and Neutrophil-to-Lymphocyte Ratio (NLR) [23]. In a study by Cetinkaya et al., it was reported that PIV has been shown to correlate with the SYNTAX score, a measure of coronary artery lesion complexity in patients with NSTEMI. Higher PIV levels were associated with more severe coronary artery lesions, suggesting that PIV could serve as a marker for assessing disease severity in CAD patients [24]. While PIV has been extensively studied in oncology for its prognostic value in cancer patients, its application in cardiology, particularly in predicting CAD outcomes, is a relatively new and promising area of research. In a study by Jing et al., they reported PIV’s efficacy in routine clinical practice for benefitting cardiovascular disease management [25]. Our study builds on these findings by integrating PIV with other novel biomarkers, such as the TGI and RAR, to provide a more comprehensive risk assessment model for CAD.

In recent years, the importance of inflammation indices in the prognosis of CAD has gained increasing attention. Besides the RDW–albumin ratio, Triglyceride–glucose index, and pan-immune-inflammation value explored in this study, other indices such as the Advanced Lung Cancer Inflammation Index (ALI) have also shown prognostic value in cardiovascular diseases. Trimarchi et al. highlighted the role of ALI as a predictor of all-cause mortality in ST-elevation myocardial infarction (STEMI) patients undergoing primary percutaneous coronary intervention. The authors found that elevated ALI levels were significantly associated with increased mortality risk, suggesting that ALI could be an additional marker for risk stratification in CAD patients [26]. Incorporating indices like ALI into clinical practice may enhance the predictive accuracy of existing models and offer a more comprehensive approach to managing high-risk patients with CAD.

### Limitations of the Study

Our study was conducted on a relatively limited sample size, which may not fully capture the diversity of the population. Conducted at a single center, our study may reflect site-specific practices and patient characteristics, which could differ from those at other institutions or regions. Although we explored a range of biomarkers, other potentially relevant biomarkers were not included in this study.

## 5. Conclusions

In conclusion, this study highlights the prognostic value of novel biomarkers—RAR, TGI, and PIV—in predicting CAD. The combined use of these biomarkers provides improved diagnostic accuracy, suggesting their potential as complementary tools alongside traditional risk factors in assessing CAD risk. While the results are promising, further studies are necessary to validate these findings in larger and more diverse patient populations. Additionally, future research should explore the clinical utility of these biomarkers in routine practice, particularly in enhancing risk stratification and guiding early interventions in high-risk CAD patients.

## Figures and Tables

**Table 1:** The comparison of sociodemographic and clinical characteristics.

Variables	Normal Group (n = 150)	CAD Group (n = 450)	*p* Value
Age (years)	65.0 ± 10.3	70.3 ± 9.8	<0.02
Male n (%)	75 (50%)	270 (60%)	<0.01
BMI (kg/m^2^)	24.6 ± 2.1	27.3 ± 3.4	<0.04
DBP (mmHg)	76.5 ± 8.7	82.1 ± 10.2	<0.03
SBP (mmHg)	121.4 ± 13.5	136.8 ± 15.3	<0.03
Heart rate	72.3 ± 7.4	78.9 ± 8.9	<0.03
Smoking n (%)	30 (20%)	180 (40%)	<0.001
Hypertension n (%)	45 (30%)	300 (67%)	<0.001
Hypercholesterolemia n (%)	50 (33%)	320 (71%)	<0.001
Ejection fraction (EF)	60 ± 5	48 ± 10	<0.001

CAD, coronary artery disease; BMI, body mass index; DBP, diastolic blood pressure; SBP, systolic blood pressure.

**Table 2:** Comparison of laboratory findings.

Variables	Normal Group (n = 150) Mean ± SD	CAD Group (n = 450) Mean ± SD	*p* Value
Hemoglobin (g/L)	140 ± 15	130 ± 20	<0.05
Leukocyte (10^9^×L^−1^)	6.0 ± 1.5	8.0 ± 2.3	<0.05
Neutrophils (10^9^×L^−1^)	3.6 ± 1.2	5.5 ± 1.6	<0.05
Lymphocytes (10^9^×L^−1^)	2.0 ± 0.5	1.5 ± 0.4	<0.05
Monocytes (10^9^×L^−1^)	0.4 ± 0.1	0.6 ± 0.2	<0.05
Platelets (10^9^×L^−1^)	250 ± 50	300 ± 75	<0.01
Glucose (mg/dL)	90 ± 15	130 ± 30	<0.01
Total cholesterol (mg/dL)	180 ± 30	220 ± 40	<0.01
HDL (mg/dL)	55 ± 12	40 ± 10	<0.01
LDL (mg/dL)	100 ± 25	150 ± 35	<0.01
Triglyceride (mg/dL)	110 ± 40	170 ± 50	<0.01
Bilirubin (µmol/L)	10 ± 3	12 ± 4	<0.05
Creatinine (µmol/L)	70 ± 20	85 ± 25	<0.05
BUN (mmol/L)	6 ± 2	8 ± 3	<0.05
RDW (%)	13 ± 2	15 ± 3	<0.01
Albumin (g/dL)	4.5 ± 0.5	4.0 ± 0.4	<0.01
RDW—albumin ratio (RAR)	3.2 ± 0.5	4.0 ± 0.7	<0.01
Triglyceride Glucose Index (TGI)	4.2 ± 0.6	5.0 ± 0.8	<0.01
Pan-Immune-Inflammation Value (PIV)	250 ± 100	500 ± 150	<0.01

CAD: Coronary artery disease. RDW: Red blood cell distribution width.

**Table 3:** Stepwise logistic regression analysis (forward method) for association of parameters with CAD.

Variables	Odds Ratio	95% CI	*p* Value
Age (years)	1.04	1.02–1.06	<0.01
Male (n%)	1.1	0.8–1.2	<0.01
BMI (kg/m^2^)	1.2	1.1–1.3	<0.05
Smoking (n%)	1.2	1.1–1.5	<0.01
Hypertension (n%)	1.5	1.2–2.2	<0.01
Hypercholesterolemia (n%)	1.3	1.6–3.1	<0.01
Diabetes mellitus (n%)	1.8	1.4–2.8	<0.01
Ejection fraction (EF)	0.9	0.7–1.4	<0.05
Neutrophil (10^9^×L^−1^)	1.1	0.9–1.6	<0.01
HDL (mg/dL)	0.9	0.6–1.2	<0.01
LDL (mg/dL)	1.3	1.1–1.6	<0.01
Triglyceride (mg/dL)	1.3	1.1–1.5	<0.01
RDW (%)	1.1	0.9–1.4	<0.05
Albumin (g/dL)	0.8	0.7–0.9	<0.05
RDW—Albumin ratio (RAR)	1.6	1.2–1.9	<0.01
Triglyceride–Glucose Index (TGI)	1.8	1.4–2.2	<0.01
Pan-Immune-Inflammation Value (PIV)	2.0	1.7–2.4	<0.01

BMI: Body mass index.

**Table 4:** ROC analysis results in patients with CAD.

Parameters	AUC (95% CI)	Sensitivity	Specificity	CUT-OFF	*p* Value
Age	0.65 (0.62–0.68)	60%	65%	65<	<0.01
BMI (kg/m^2^)	0.67 (0.64–0.70)	63%	68%	27.5<	<0.01
Smoking	0.70 (0.67–0.73)	65%	70%	-	<0.01
Hypertension	0.72 (0.69–0.75)	68%	72%	-	<0.01
Diabetes mellitus	0.74 (0.71–0.77)	70%	74%	-	<0.01
Hypercholesterolemia	0.68 (0.65–0.71)	66%	68%	-	<0.01
RDW (%)	0.66 (0.63–0.69)	64%	66%	14.5	<0.01
RDW–Albumin ratio (RAR)	0.70 (0.67–0.73)	68%	70%	3.8<	<0.01
Triglyceride–Glucose Index (TGI)	0.71 (0.68–0.74)	69%	71%	9.0	<0.01
PIV	0.75 (0.72–0.78)	72%	75%	368.8<	<0.01
Combination of RAR, TGI, and PIV	0.78 (0.75–0.81)	74%	77%	-	<0.01

BMI: Body mass index. PIV: Pan-immune-inflammation.

## Data Availability

The original contributions presented in the study are included in the article. Further inquiries can be directed to the corresponding author.

## References

[1] Tsao C.W., Aday A.W., Almarzooq Z.I., Anderson C.A.M., Arora P., Avery C.L., Baker-Smith C.M., Beaton A.Z., Boehme A.K., Buxton A.E. (2023). Heart Disease and Stroke Statistics-2023 Update: A Report From the American Heart Association. Circulation.

[2] Malakar A.K., Choudhury D., Halder B., Paul P., Uddin A., Chakraborty S. (2019). A review on coronary artery disease, its risk factors, and therapeutics. J. Cell. Physiol..

[3] Ni Q., Wang X., Wang J., Chen P. (2022). The red blood cell distribution width-albumin ratio: A promising predictor of mortality in heart failure patients—A cohort study. Clin. Chim. Acta.

[4] Galindo-Martin C.A., Chong-Avina P.A., Godinez-Breacher V., Aportela-Vazquez V.A., Bueno-Hernandez G., Gante-Garcia M.F., Pimentel-Luna K.Y., Sanchez-Abrego M. (2024). Malnutrition: Muscle wasting, inflammation, RDW, and their relation with adverse outcomes. Cir. Cir..

[5] Manolis A.A., Manolis T.A., Melita H., Mikhailidis D.P., Manolis A.S. (2022). Low serum albumin: A neglected predictor in patients with cardiovascular disease. Eur. J. Intern. Med..

[6] Wang L., Wang Y., Liu R., Xu L., Zhong W., Li L., Wang C., He C., Fu C., Wei Q. (2022). Influence of age on the association between the Triglyceride–glucose index and all-cause mortality in patients with cardiovascular diseases. Lipids Health Dis..

[7] Cardenas-Juarez A., Portales-Perez D.P., Rivas-Santiago B., Garcia-Hernandez M.H. (2024). Clinical Significance of the Lipid Profile Ratios and Triglyceride Glucose Index in the Diagnosis of Metabolic Syndrome. Metab. Syndr. Relat. Disord..

[8] Yi C., Zhou Y.N., Guo J., Chen J., She X. (2024). Novel predictors of intravenous immunoglobulin resistance in patients with Kawasaki disease: A retrospective study. Front. Immunol..

[9] Liu Y., Liu J., Liu L., Cao S., Jin T., Chen L., Wu G., Zong G. (2023). Association of Systemic Inflammatory Response Index and Pan-Immune-Inflammation-Value with Long-Term Adverse Cardiovascular Events in ST-Segment Elevation Myocardial Infarction Patients After Primary Percutaneous Coronary Intervention. J. Inflamm. Res..

[10] Feng Y.-M., Kim J.H., Lim S., Park K.S., Jang H.C., Choi S.H. (2017). Total and differential WBC counts are related with coronary artery atherosclerosis and increase the risk for cardiovascular disease in Koreans. PLoS ONE.

[11] Luo J., Thomassen J.Q., Nordestgaard B.G., Tybjærg-Hansen A., Frikke-Schmidt R. (2023). Neutrophil counts and cardiovascular disease. Eur. Heart J..

[12] Sun X., Fan Z., Liu Z., Li J., Hua Q. (2022). Red blood cell distribution width-to-albumin ratio: A new inflammatory biomarker to predict contrast-induced nephropathy after emergency percutaneous coronary intervention. Int. Urol. Nephrol..

[13] Gu Y.-l., Yang D., Huang Z.-b., Chen Y., Dai Z.-s. (2022). Relationship between red blood cell distribution width-to-albumin ratio and outcome of septic patients with atrial fibrillation: A retrospective cohort study. BMC Cardiovasc. Disord..

[14] Huang M., Liu F., Li Z., Liu Y., Su J., Ma M., He Y., Bu H., Gao S., Wang H. (2023). Relationship between red cell distribution width/albumin ratio and carotid plaque in different glucose metabolic states in patients with coronary heart disease: A RCSCD-TCM study in China. Cardiovasc. Diabetol..

[15] Hong J., Hu X., Liu W., Qian X., Jiang F., Xu Z., Shen F., Zhu H. (2022). Impact of red cell distribution width and red cell distribution width/albumin ratio on all-cause mortality in patients with type 2 diabetes and foot ulcers: A retrospective cohort study. Cardiovasc. Diabetol..

[16] Li H., Xu Y. (2023). Association between red blood cell distribution width-to-albumin ratio and prognosis of patients with acute myocardial infarction. BMC Cardiovasc. Disord..

[17] Li D., Long J., Zhang J., He M., Zeng Q., He Q., Zhan W., Chi Y., Zou M. (2024). Association between red cell distribution width–and–albumin ratio and the risk of peripheral artery disease in patients with diabetes. Front. Endocrinol..

[18] Xiong S., Chen Q., Long Y., Su H., Luo Y., Liu H., Chen Y., Feng Q., Peng X., Jiang M. (2023). Association of the triglyceride–glucose index with coronary artery disease complexity in patients with acute coronary syndrome. Cardiovasc. Diabetol..

[19] Liang S., Wang C., Zhang J., Liu Z., Bai Y., Chen Z., Huang H., He Y. (2023). Triglyceride–glucose index and coronary artery disease: A systematic review and meta-analysis of risk, severity, and prognosis. Cardiovasc. Diabetol..

[20] Zhao S., Wang Z., Qing P., Li M., Liu Q., Pang X., Wang K., Gao X., Zhao J., Wu Y. (2024). Comprehensive analysis of the association between Triglyceride–glucose index and coronary artery disease severity across different glucose metabolism states: A large-scale cross-sectional study from an Asian cohort. Cardiovasc. Diabetol..

[21] Gao A., Liu J., Hu C., Liu Y., Zhu Y., Han H., Zhou Y., Zhao Y. (2021). Association between the triglyceride glucose index and coronary collateralization in coronary artery disease patients with chronic total occlusion lesions. Lipids Health Dis..

[22] Tao S., Yu L., Li J., Huang L., Huang X., Zhang W., Xie Z., Tan Y., Yang D. (2023). Association between the Triglyceride–glucose index and 1-year major adverse cardiovascular events in patients with coronary heart disease and hypertension. Cardiovasc. Diabetol..

[23] Dolapoglu A., Avci E. (2024). Relationship between pan-immune- inflammation value and in major cardiovascular and cerebrovascular events in stable coronary artery disease patients undergoing on-pump coronary artery bypass graft surgery. J. Cardiothorac. Surg..

[24] Cetinkaya Z., Kelesoglu S., Tuncay A., Yilmaz Y., Karaca Y., Karasu M., Secen O., Cinar A., Harman M., Sahin S. (2024). The Role of Pan-Immune-Inflammation Value in Determining the Severity of Coronary Artery Disease in NSTEMI Patients. J. Clin. Med..

[25] Hai-Jing Y., Shan R., Jie-Qiong X. (2023). Prognostic significance of the pretreatment pan-immune-inflammation value in cancer patients: An updated meta-analysis of 30 studies. Front. Nutr..

[26] Trimarchi G., Pizzino F., Lilli A., De Caterina A.R., Esposito A., Dalmiani S., Mazzone A., Di Bella G., Berti S., Paradossi U. (2024). Advanced Lung Cancer Inflammation Index as Predictor of All-Cause Mortality in ST-Elevation Myocardial Infarction Patients Undergoing Primary Percutaneous Coronary Intervention. J. Clin. Med..

